# Evaluation of Micro-Tensile Bond Strength of Fibre Post with Titanium Dioxide Nanoparticles as Fillers in Experimental Dental Composite Resin

**DOI:** 10.3390/ma15093312

**Published:** 2022-05-05

**Authors:** Dhanasekaran Sihivahanan, Mavishna Maniyan Vijayakumari, Pradeep Kumar Yadalam, Nezar Boreak, Sultan Binalrimal, Saeed M. Alqahtani, Mohammed Hussain Dafer Al Wadei, Thilla Sekar Vinothkumar, Hitesh Chohan, Harisha Dewan, Shilpa Bhandi, Shankargouda Patil

**Affiliations:** 1 Department of Conservative Dentistry and Endodontics, SRM Kattankulathur Dental College and Hospital, SRM Institute of Science and Technology, Chennai 603203, India; shivahad@srmist.edu.in; 2 Department of Conservative Dentistry and Endodontics, Mahatma Gandhi Postgraduate Institute of Dental Sciences, Puducherry 605006, India; mavishna11@gmail.com; 3 Department of Periodontics, Saveetha Dental College and Hospitals, Saveetha Institute of Medical and Technical Sciences, Saveetha University, Chennai 600077, India; pradeepkumar.sdc@saveetha.com; 4 Department of Restorative Dental Sciences, College of Dentistry, Jazan University, Jazan 45142, Saudi Arabia; nezarboreak@gmail.com (N.B.); vinothkumar_ts@yahoo.com (T.S.V.); drhiteshchohan@yahoo.co.in (H.C.); shilpa.bhandi@gmail.com (S.B.); 5 Department of Restorative Science, College of Dentistry, Riyadh Elm University, Riyadh 12611, Saudi Arabia; sultan@riyadh.edu.sa; 6 Department of Prosthetic Dentistry, College of Dentistry, King Khalid University, Abha 61421, Saudi Arabia; smaalqahtani@kku.edu.sa; 7 Department of Restorative Dental Science, King Khalid University, Abha 61421, Saudi Arabia; moalwadai@kku.edu.sa; 8 Department of Conservative Dentistry and Endodontics, Saveetha Dental College, Saveetha Institute of Medical and Technical Sciences, Chennai 600077, India; 9 Department of Prosthetic Dental Sciences, College of Dentistry, Jazan University, Jazan 45142, Saudi Arabia; harisha.dewan@yahoo.com; 10 Department of Maxillofacial Surgery and Diagnostic Sciences, Division of Oral Pathology, College of Dentistry, Jazan University, Jazan 45142, Saudi Arabia

**Keywords:** adhesion, bond strength, composite core, FRC post, nanoparticles, titanium dioxide

## Abstract

Background: The clinical success of post-core restorations is determined by the composite utilized and the strength of the post-core adhesion. The effectiveness of titanium dioxide nanoparticles (TiO_2_ NPs) as a multifunctional material with photo-induced activities and better mechanical characteristics are observed as particle size is reduced to under 50 nm. Aim: The purpose of this study is to determine the bond strength of fibre-reinforced composite (FRC) posts with TiO_2_ NP as fillers and to compare it with conventional composite resin core material. Materials and Methods: 30 single-rooted mandibular premolars were selected and routine root canal procedures were done. A quantity of 5% TiO_2_ NPs were synthesized and added as silanized filler to the experimental composite resin. Post space was prepared and fibre-reinforced composite (FRC) post luting was performed. The specimens were then grouped into the following groups: Group I consisted of the experimental composite resin containing 5% TiO_2_ fillers, Group II consisted of core X flow, and Group III consisted of Multicore Flow. All test groups were submitted for thermocycling. After this, the samples were tested for micro tensile bond strength. A stereomicroscope with a magnification of 20× was used to examine the fractured surfaces. The data were analysed using one-way ANOVA and Tukey HSD tests. Results: Statistical analysis revealed that Group I showed the highest mean bond strength value of 35.6180 Mpa. The results obtained with Group III showed the lowest mean bond strength value of 19.4690 Mpa. Adhesive failures were identified by stereomicroscopy of the fractured surfaces. Conclusion: The experimental composite resin comprising 5% TiO_2_ NP had a greater bond to the FRC post than other materials tested.

## 1. Introduction

A cast metal post has traditionally been used to retain an endodontically treated tooth to facilitate prosthetic rehabilitation once the treatment has been completed. Since their introduction in the 1990s, fibre posts have been suggested as a possible substitute to cast metal posts for endodontically treated tooth restoration [1]. Fibre posts have many advantages over metal posts, including improved aesthetics, better dentin bonding, corrosion resistance, and a decreased occurrence of vertical root fracture. Although posts do not reinforce endodontically treated teeth, it was observed that they do play a vital role in maintaining a core when significant parts or the entire clinical crown are lost [2].

A core build-up is a reconstruction used to repair the majority of the coronal portion of a tooth that has been badly damaged [3]. The ideal core materials should provide favourable stress distribution of forces, thereby minimizing the tensile and compressive fractures. Composite scores are popular because of their command set and retentive stability. The modulus of elasticity should be equal to or higher than that of dentine [4].

The micro-tensile bond strength test (μ-TBS) is the best surrogate measure of dental composite restoration retention [5]. Although many techniques are available to evaluate the bond strength, the μ-TBS allows the researchers to emphasize more the clinically relevant substrates with three-dimensional surfaces [6].

Titanium dioxide nanoparticles (TiO_2_ NPs) are non-toxic, chemically inert, have a high refractive index, have broad-spectrum antibacterial capabilities, are resistant to corrosion, and have a high hardness. Because of their remarkable photoactivity and exceptional mechanical qualities, TiO_2_ NPs are one of the greatest additions for increasing the performance of polymeric materials [7,8]. Current literature research did not reveal the bond strength of TiO_2_ NP as fillers in the composite resin with the fibre-reinforced composite (FRC) post.

Thus, the objective of this study is to ascertain the bond strength of fibre posts with titanium dioxide nanoparticles as fillers in experimental dental composite resin and to compare it to the bond strength of traditional composite resins in use as core materials, namely Multicore Flow and Core X Flow.

## 2. Materials and Methods

The study was conducted at the SRM Institute of Science and Technology’s Nanotechnology Research Department, ethical clearance number: 1798/IEC/2019.

### 2.1. Titanium Dioxide Nanoparticle Synthesis

To synthesize 5% of TiO_2_ NP, the technique previously described by Venkatasubbu et al. (2012) was utilized. The characterization of the TiO_2_ NP and the uniform distribution of the nanoparticles were analysed using samples for transmission electron microscopy (TEM, JEOL, Tokyo, Japan). The TEM analysis was undertaken by placing TiO_2_ nanoparticles onto a carbon-coated copper grid that had been left at room temperature overnight. The images were captured using a Philips EM400T operating at 200 kV, with a magnification of roughly 50 nanometres (nm) and a point-to-point resolution of 2 A° [9].

### 2.2. Synthesis of an Experimental Composite Using Titanium Dioxide as a Filler

The monomer synthesis and silanization of filler particles, as well as the fabrication of the experimental composite resin, were done using the technique previously published by Sihivahanan et al. (2021) [10].

The monomer matrix was composed of bisphenol A glycidyl methacrylate (bis-GMA) and triethylene glycol dimethacrylate (TEGDMA). In addition, diketone was employed as a photoinitiator, and N, N-dimethylaminoethyl methacrylate (DMAEMA) was used as a co-initiator in matrix preparation. All of the ingredients were acquired from Sigma-Aldrich in St. Louis, MO, USA.

Preheating BisGMA to 500 °C for 60 min in a glass container made handling the substance easier. Amber glass vials were then used to contain the monomer TEGDMA to prevent the photoinitiator from activating (500 mL, Sigma-Aldrich, St. Louis, MO, USA). A magnetic stirrer was then used to add 0.5 percent weight CQ and 0.5 percent weight DMAEMA to the monomer solution, which was then mixed for 60 min. It was then packaged in amber bottles with aluminium foil on top to protect the freshly mixed monomer from light. All components were weighed with a digital scale (0.01 g readability) using a digital scale (PERCISION Advanced: OHAUS, Troy Hills, NJ, USA).

Two types of reinforcing fillers were used: 10% silanized amorphous silica and 55% silanized aluminium silicate (Evonic Industries, Essen, Germany). Additionally, TiO_2_ NP was used as a filler at a concentration of 5%. At room temperature, the reinforcing fillers were mixed into a matrix in 50 mL crystal Griffin form beakers. An alkoxy-terminated silanating agent (1.0 vol-% of 3-methacryloxypropyltrimethoxysilane (Sigma Aldrich, St. Louis, MO, USA) was used as silane coupling agent to silanize amorphous silica, aluminium silicate fillers, and titanium dioxide nanoparticles (TiO_2_ NP).

The silane treatment of the fillers was prepared by the modified method (Sousa et al., 2003). Amorphous silica and aluminium silicate fillers were silanated using alkoxy-terminated silanating agents. A 1.0 vol-% of 3-methacryloxypropyltrimethoxysilane (Sigma Aldrich, St. Louis, MO, USA) solution was prepared using a pre-prepared solvent mixture of 90 vol-% ethanol and 10 vol-% deionised water. The pH of the solvent mixture was adjusted to 4 by 3.0 M acetic acid. The silane solution was next stirred and allowed to hydrolyse (activate) for 1 h. The filler, silanizing agent and a ketonic solvent were placed in a glass vessel. The content was stirred for 5 to 8 h at 40 to 50 °C, then the solvent was decanted off and the filler dried at 105 deg C for 2–3 h and sieved before use in the composite. Fillers were added and dispersed by ultrasonication for 15 min. Following this, the reaction mixture was stirred for 24 h at room temperature. After the silane grafting process, the reaction mixture was filtered and rinsed with absolute ethanol to remove physically adsorbed silanes. The powder was dried overnight at room temperature and then dried at 60 °C in an oven for 72 h to enhance the condensation of surface silanol molecules and to remove any remaining solvent.

The ingredients were weighed and placed in a mortar and pestle in the correct sequence. To make a composite mass, the mass was manually mixed and heated in the oven overnight between 40 to 50 °C. Approximately an hour after it was wetted at 40 to 50 °C for 24 h, it was manually blended in the mortar and held in the oven at 40 to 50 °C. This process was performed a further five to seven times or until the required consistency was attained.

### 2.3. Sample Preparation

A total of 30 freshly extracted single-rooted mandibular premolars for orthodontic purposes were collected. To prevent dehydration, a saline solution was used to preserve the samples until they were used.

The experimental procedure was done by a single investigator. The samples were examined under a stereomicroscope to evaluate them for any external cracks or defects. The samples were then decoronated at a level of 2 mm above the cementoenamel junction (CEJ) to aid in simulating the ferrule effect, which protects the tooth from fracture.

Access openings were made using a round diamond point bur; cleaning and shaping were performed using K files ranging from no. 15 to no. 50 with 5.25% sodium hypochlorite solution used as irrigating solution. Obturation was completed using gutta-percha and zinc oxide eugenol as a sealer using the lateral compaction technique. For the complete setting of the sealer, the samples were kept idle for two weeks.

Around the coronal tooth structure, a shoulder finish line with a width of 1.5 mm was formed. Peso reamers of size no. 1 to 5 (Mani, Tokyo, Japan) were used to prepare the post space. As per the researcher’s recommendation, 4 to 5 mm of gutta-percha was left from the apex.

A fibre-reinforced composite (FRC) (FRC Postec Plus, Ivoclar Vivadent, Schaan, Liechtenstein) post of size 2 with a diameter of 1.5 mm was selected. The post was trimmed to 11 mm in length, leaving 2 mm coronally protruding. Using a micro brush, a thin layer of Monobond N (Ivoclar Vivadent) was applied to the FRC post. Ivoclar Vivadent Multilink Speed resin luting cement was mixed based on the manufacturer’s (Ivoclar Vivdent, New York, NY, USA) instructions and applied to the FRC post and the prepared post area. Once inserted evenly, they were light-cured for 40 s. The samples were randomly assigned to the following groups for core cementation (Table 1).

Group I: The experimental composite resin with 5% of TiO_2_ fillers. (N = 10)

Group II: Core X Flow (Dentsply Sirona) (N = 10)

Group III: Multicore flow (Ivoclar Vivadent) (N = 10)

Two coatings of Tetric N (Ivoclar Vivadent) bond were applied to the post and light-cured for 20 s before the core build-up procedure.

**Figure 1:**
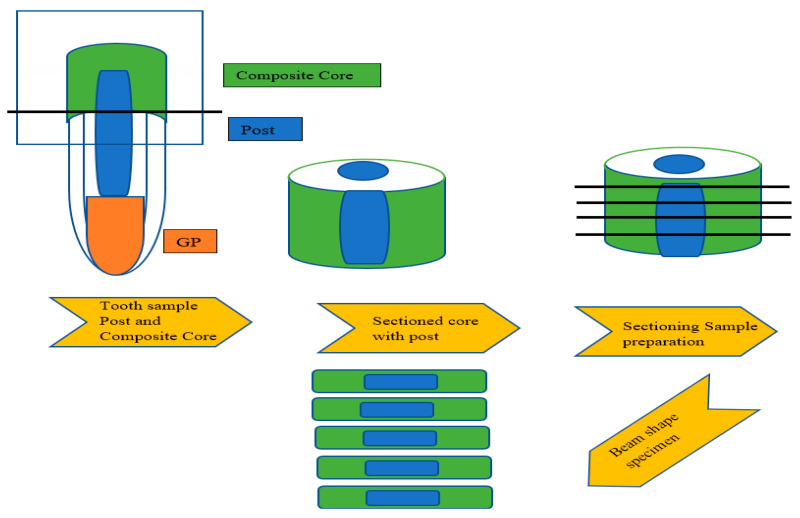
Schematic representation of samples prepared for micro-tensile bond strength.

The core was fabricated using a prefabricated core former template having dimensions of 2 mm in length and 4 mm in diameter (Figure 1). Each surface was light-cured for 20 s after the composite resin core material from each group was dispensed and condensed into the prepared matrix. The hardened core was then removed from the template. All the samples were submitted to thermocycling in deionized water baths for 15 s, at 5–55° for 5000 cycles.

Universal testing equipment (EZ Test, Shimadzu Co., Kyoto, Japan) with a crosshead speed of 0.5 mm/min was used to test the samples for microtensile bond strength according to the methods provided by Khamverdi et al. (2011). The prepared samples for microtensile bond strength were around 0.5 to 1.0 mm thick (Figure 1) [2].

By dividing the specimen’s failure load (N) by its surface area, the microtensile bond strength was determined in Mpa. The data were analysed using the one-way ANOVA followed by Tukey HSD tests. The degree of confidence was set at 95 percent for all tests. The fractured surfaces were magnified 20 times using a stereomicroscope (Nikon Eclipse E600, Tokyo, Japan). The three types of failures observed were cohesive breakdowns (failure within the post and core material), adhesive failures (failure in between the post and the core material), and hybrid failures (failure between the post and the core material).

Data regarding the microtensile bond strength of three composite resin core materials on FRC post were entered into Microsoft Excel and analysed using IBM SPSS Statistics for Windows, Version 20 (IBM Corp., Armonk, NY, USA). Data were explored for normality using the Shapiro–Wilk test. Descriptive statistics and microtensile bond strength across three groups were analysed using a one-way analysis of variance (ANOVA), followed by multiple comparisons using Tukey’s honest significant difference test (α = 0.05). The level of statistical significance was determined at *p* ≤ 0.05.

## 3. Results

**Figure 2:**
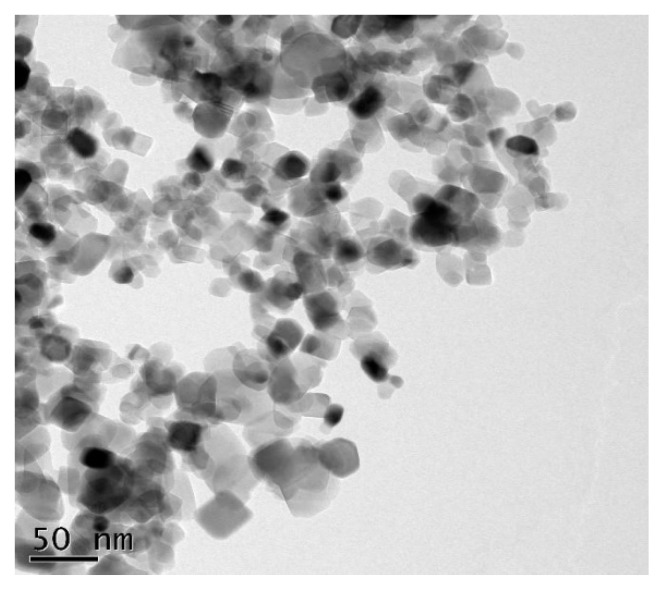
TiO_2_ NP under TEM with a magnification of roughly 50 nanometres.

The transmission electron microscope (TEM) analysis of the TiO_2_ NP showed a spherical smooth shaped uniform distribution of the nanoparticles. The nanoparticles were in the range of 250–300 nm scale (Figure 2—TiO_2_ NP under TEM with a magnification of roughly 50 nanometres).

Table 2 shows the mean and standard deviations of microtensile bond strength for the three groups. Group I showed a mean bond strength value of 35.6180 Mpa. Group II showed a mean bond strength value of 24.4040 Mpa. Group III showed a mean bond strength value of 19.4690 Mpa.

Table 3 shows the post hoc comparison analysis between the groups. The mean bond strength difference between Group I and Group II was 11.2 Mpa and the mean bond strength difference between Group I and Group III was 16.14 Mpa. The bond strength difference between Group II and Group III was 4.9 Mpa.

**Figure 3:**
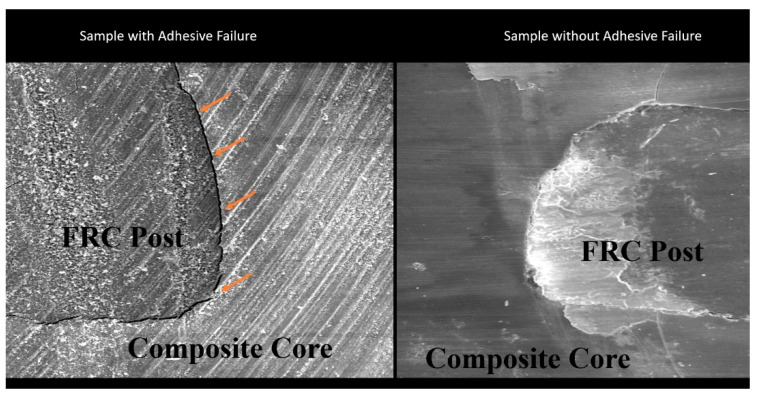
Stereomicroscopic image with 20× magnification of adhesive failure.

The broken surfaces were examined stereomicroscopically, and it was discovered that in all the groups the majority of failures were due to adhesive failures seen between the post and the core material (Figure 3).

The usage of resin composites as core materials has a substantial impact on microtensile bond strength (*p* ≤ 0.05), according to statistical analysis. The Group 1 experimental composite with 5% TiO_2_ shows a higher bond strength to the FRC post than the other materials compared.

## 4. Discussion

The prognosis of endodontic treatment is contingent upon both the effectiveness of the endodontic procedure and the post endodontic reconstruction with an ideal core material. The core material must maintain functional loadings by minimizing stress concentrations at the tooth/post interface. If the adhesion at these interfaces of the core material is weak, then there is a higher incidence of debonding of post and core leading to a catastrophic failure [1,11,12].

As a core material, the material should have a high strength to resist fracture and good bond strength to the post as well as to the residual tooth structure for it to function. A previous study on incorporating TiO_2_ NP in composite resin has shown improved mechanical properties compared to traditional composite resin [10]. Hence, in this study, the TiO_2_ NP was incorporated to evaluate the bondable property of the experimental composite resin core material.

Silane was applied to the post before luting with the resin cement. The silane coupling agent will improve the adherence of the resin cement with the post layer. Because of their low viscosity, silane coupling agents aid in substrate wetting. If an adequate interaction occurs between the interface materials, van der Waals forces kick in, resulting in a physical adhesion that might aid chemical interactions [13].

Bond strength can be evaluated using a variety of mechanical measures. Since multiple specimens can be collected from a single tooth, the microtensile bond strength (μ-TBS) is more flexible, allowing for more creative set-ups and better control of substrate variables [14].

This study’s objective was to determine the bond strength of fibre-reinforced composite (FRC) posts with 5% TiO_2_ NP as fillers in experimental dental composite resin and to compare it with traditional composite resin core material. Two conventional core materials were chosen as the comparative groups from a spectrum of composite resin core materials currently available, which were Core X Flow from Dentsply Sirona and Multicore Flow from Ivoclar Vivadent. The rationale for selecting the material for the comparative group is to evaluate the effect of the type of filler and the filler percentage on the bond strength with the FRC post.

Based on the results from previous studies, the percentage of TiO_2_ as fillers in the experimental composite resin was selected as 5%. Studies have shown that increasing the percentage of fillers above 5% results in the loss of bond strength with the tooth structure [15].

Table 2 shows the result of mean and SD values of the microtensile bond strength test in Mpa. Group I (experimental composite resin with 5% of TiO_2_) showed a higher mean microtensile bond strength of 35.61 Mpa. Table 3 shows the difference between the microtensile bond strength of composite core material. The difference between Group I and Group II was 11.21400 Mpa and the difference between Group I and Group III was 16.14900 Mpa. The results of the study indicate that the 5%TiO_2_ fillers performed significantly better than the Core X Flow, followed by the Multicore Flow.

Based on the analysis done by Xia et al. (2008) on the TiO_2_ NP, it has been shown that the nano-TiO_2_ particles are encased with an organosilane to improve their adhesion to the resin matrix and extend the bond strength of the composite core material [16].

Ziental et al. (2020) have shown that the photochemical ability of titanium dioxide help in the polymerization reaction. Becker-Willinger et al. (2010) have also proven in their research that the TiO_2_ nanoparticles can be used as photosensitive initiators to produce free radical polymerization. Raorane et al. (2019) in their research and based on studies done by Rastelli et al. (2012) have shown that 5% TiO_2_ NP lowers the polymerization shrinkage [17,18,19].

The increased bond strength with 5% TiO_2_ NP can be due to the complete polymerization of the resin matrix. The increased polymerization led to decreased polymerization contraction and in turn improved the bond strength. This resultant increased bond strength is due to the unique photochemical activity along with the silanization of TiO_2_ NP.

The results of this study with regards to the comparative groups of the dual-cured flowable composite resins follow a previous study by Asim Al-Ansari et al. (2015). In their study, the evaluated dual-polymerized core foundations, when implemented with chemically polymerized adhesives, had lower bond strengths. These data could imply a reduced degree of adhesive polymerization due to a lack of peroxide/amine concentration—the less chemical activator/initiator, the lower the degree of adhesive polymerization [20].

The evaluation of the comparative groups shows that Group II (Core X Flow) had a mean microtensile bond strength of 24.40 Mpa, which is lower than the experimental composite core material but higher than Group III (Multicore Flow). The difference between Group II and Group III was 4.93500 Mpa, which shows that the Core X Flow performed better than the Multicore Flow.

The Core X Flow composite resin showed a better bond strength value than the Multicore Flow. The Core X Flow composite resin has 70 wt% filler content. Condon et al. (2000) and Baroudi et al. (2007) in their studies have concluded that there is a relationship between filler content and contraction stress and it has already been established that densely filled composites exhibit lower volumetric shrinkage and exhibit less contraction stress. Koçak MM et al. (2012) have also concluded in their study that the composites with a higher filler content will have less shrinking. According to this hypothesis, the highest bond strength for fibre posts was found in light-core composites with greater filler content. The findings of this investigation were consistent with the findings of a previous study by Srinu et al. (2020), who concluded in their research that the filler concentration and density of filler particles were higher in specimens with Core X Flow as the core material, at 1.95 g per centimetre cubed. The tightly packed fillers on the resulting surface aid in better bonding with the tooth structure [21,22,23].

The Multicore Flow composite resin has the lowest bond strength values. The filler content of Multicore Flow composite resin is 55 wt%, which is lower than the Core X Flow composite resin.

Although the filler content of the Multicore Flow is lower, it can be used as a core material. The findings of this investigation match those of a previous study by Sadek et al. (2005); in their study it was shown that the flowable composites showed good flexibility at the post surface due to their low viscosity, making them suitable options for core build-up materials. Since the filler content is less, there are decreased bond strength values. This was conformed with the findings of a study conducted by Bayne et al. (1998), who have shown that the high resinous concentration of these materials might cause substantial contraction during polymerization. The interfacial bond can be weakened by shrinkage-strain stress, decreasing the strength of the bond to the post surface [1].

All the samples used in the study had an adhesive failure. The bond failure occurred between the FRC post and the core composite material. This was in accordance with the study done by Al-Ansari et al. (2015), who has concluded in their study that the chemically or dual-polymerized adhesive layer did not offer enough reactive resin radicals for the foundation resin to form a good bond, and hence the adhesive failure. Research done by Koçak MM et al. (2012) has also shown adhesive failure could be attributable to the weakened interfacial bond by shrinkage strain, and hence the decreased bond strength to the post surface [20,22].

The current study investigated a packable experimental composite resin in comparison with commercial variants of dual-cured flowable composite resin, used as the core in a “post and core” set-up. However, the results of this study could have been more distinctive if the experimental composite resin had been compared to packable composite resins rather than the flowable composite ones. Because all of the samples failed to adhere, comparing different adhesives with different etching processes may yield different results.

## 5. Conclusions

Within the limitations of the investigation, it can be inferred that the experimental composite resin containing 5% TiO_2_ NP has a stronger bond to the FRC post than the other materials studied, and the Multicore Flow composite resin has the weakest bond to the FRC post.

## Figures and Tables

**Table 1:** Composition of composite resin used in this study.

Group	Composite Resin	Composition	Percentage of Filler
Group 1	Experimental composite resin with 5% of TiO_2_	BisGMA, TEGDMA, aluminium silicate fillers, TiO_2_, diketone photoinitiator, DMAEMA, UV stabilizer.	Aluminium silicate—55 wt% Amorphous silica—10 wt% Titanium dioxide—5 wt%
Group 2	Core X Flow	EBPADMA urethane resin, urethane dimethacrylate resin, trimethylolpropane trimethacrylate, 2,2′-ethylendioxydiethyldimethacrylat, dibenzoyl peroxide	70 wt%
Group 3	Multicore flow	Dimethacrylate, barium glass, fillers, Ba-Al-fluorosilicate glass, silicon dioxide, ytterbium trifluoride, catalysts, stabilizer, pigments	Base: 54.9 wt% Catalyst: 54.4 wt%

**Table 2:** Microtensile bond strength in Mpa of three composite resin core materials among the study groups.

Microtensile Bond Strength in Mpa	N	Mean	Std. Deviation
Group I	10	35.6180	1.14364
Group II	10	24.4040	1.00244
Group III	10	19.4690	0.61122

**Table 3:** Post hoc analysis of microtensile bond strength of core material between three different composite resins (Tukey HSD).

(I) Groups	(J) Groups	Mean Difference (I–J)	Std. Error	*p*-Value
1	2	11.21400 *	0.42319	0.000
	3	16.14900 *	0.42319	0.000
2	1	−11.21400 *	0.42319	0.000
	3	4.93500 *	0.42319	0.000
3	1	−16.14900 *	0.42319	0.000
	2	−4.93500 *	0.42319	0.000

* Mean difference is significant at the 0.05 level.
